# Dynamical visualization of anisotropic electromagnetic re-emissions from a single metal micro-helix at THz frequencies

**DOI:** 10.1038/s41598-020-80510-y

**Published:** 2021-02-08

**Authors:** T. Notake, T. Iyoda, T. Arikawa, K. Tanaka, C. Otani, H. Minamide

**Affiliations:** 1 Center for Advanced Photonics, RIKEN, 519-1399, Aramaki-aza Aoba, Sendai, 980-0845 Japan; 2 Harris Science Research Institute, Doshisha University, 1-3 Tatara Miyakodani, Kyotanabe, Kyoto 610-0394 Japan; 3 Department of Physics, Graduate School of Science, Kyoto University, Kitashirakawa Oiwake-cho, Sakyo-ku, Kyoto, 606-8502 Japan

**Keywords:** Optics and photonics, Optical materials and structures

## Abstract

The capability for actual measurements—not just simulations—of the dynamical behavior of THz electromagnetic waves, including interactions with prevalent 3D objects, has become increasingly important not only for developments of various THz devices, but also for reliable evaluation of electromagnetic compatibility. We have obtained real-time visualizations of the spatial evolution of THz electromagnetic waves interacting with a single metal micro-helix. After the micro-helix is stimulated by a broadband pico-second pulse of THz electromagnetic waves, two types of anisotropic re-emissions can occur following overall inductive current oscillations in the micro-helix. They propagate in orthogonally crossed directions with different THz frequency spectra. This unique radiative feature can be very useful for the development of a smart antenna with broadband multiplexing/demultiplexing ability and directional adaptivity. In this way, we have demonstrated that our advanced measurement techniques can lead to the development of novel functional THz devices.

## Introduction

Metal helices have played significant roles as chiral elements since Lindman first found optical rotation of radio waves at 0.9–2.5 GHz^1^. His centimeter-sized metal helices behave as optical active molecules for radio-waves, analogous to the established picture of optical activity arising from asymmetric molecular structures. This work has led to further careful investigations that consider the circular dichroism and anisotropic scattering of electromagnetic waves^2–7^. Metal helices have stepped into the limelight as basic inclusions that exhibit electromagnetic coupling in bi-isotropic materials, and more generally they have been included in bi-anisotropic metasurfaces for exotic wave manipulations^8^. Sub-wavelength-scale metal helices are required in huge quantities as inclusions to exert the functions. However, the situation becomes increasingly severe as the frequency of the electromagnetic waves increases, even for laboratory prototypes, and it becomes worse in practical manufacturing applications because of the minimum limits of machine processing (ca. 50 μm in diameter).

Kamata et al. have developed a unique process for mass-production of metal helices smaller than the machinable limit by employing electroless plating on the biological algae *Spirulina*^9^. Billions of metal-coated helices, about 30 μm in diameter and 100–300 μm in length, can be manufactured from a 10-L culture in one week. The helical structure of *Spirulina* can be controlled by changing incubator conditions such as temperature and illumination intensity during photosynthesis. In addition, various kinds of metal coatings are possible. By using the smallest metal helices as inclusions in a paraffin sheet, circular dichroism has been observed in the terahertz (THz) electromagnetic-wave region, providing a long-awaited update to Lindman’s work. Despite the tiny concentration of the metal helices (1–2 wt%, corresponding to 10^10^ helices/L), they drastically attenuate THz electromagnetic waves (e.g. − 30 dB/mm at 0.4 THz)^9^. If such strong attenuation is due to intrinsic absorption by individual helices acting as micrometer-sized “optically active molecules,” an anomalously large molar extinction coefficient of 10^11^ L/mol/cm is obtained. This is a million times larger than the attenuation produced by strongly colored dye molecules such as chlorophyll. Considering all the ohmic, dielectric, and radiative losses is an essential to clarify the mechanism responsible for this anomalous attenuation. Furthermore, chain reactions of re-absorptions and re-emissions from the excited helices may accumulate in the sheet, which might explain the strong attenuation. In any case, to measure how a single metal helix interacts with THz electromagnetic waves is a first step toward understanding the phenomenon and is the motivation for this study.

We show through movies how a single metal micro-helix, obtained from a biological *Spirulina* template, responds electromagnetically to impulsive irradiation of THz electromagnetic waves with tens of femtoseconds on a 2D scale of micrometers resolutions. We utilized a THz near-field microscope to visualize the spatial evolution of the THz electric fields in real time. Our findings contribute not only to understanding the electromagnetic response of a single metal micro-helix, but also the development of various devices for a future highly-advanced wireless society surrounded by THz electromagnetic waves.

## Results

**Figure 1:**
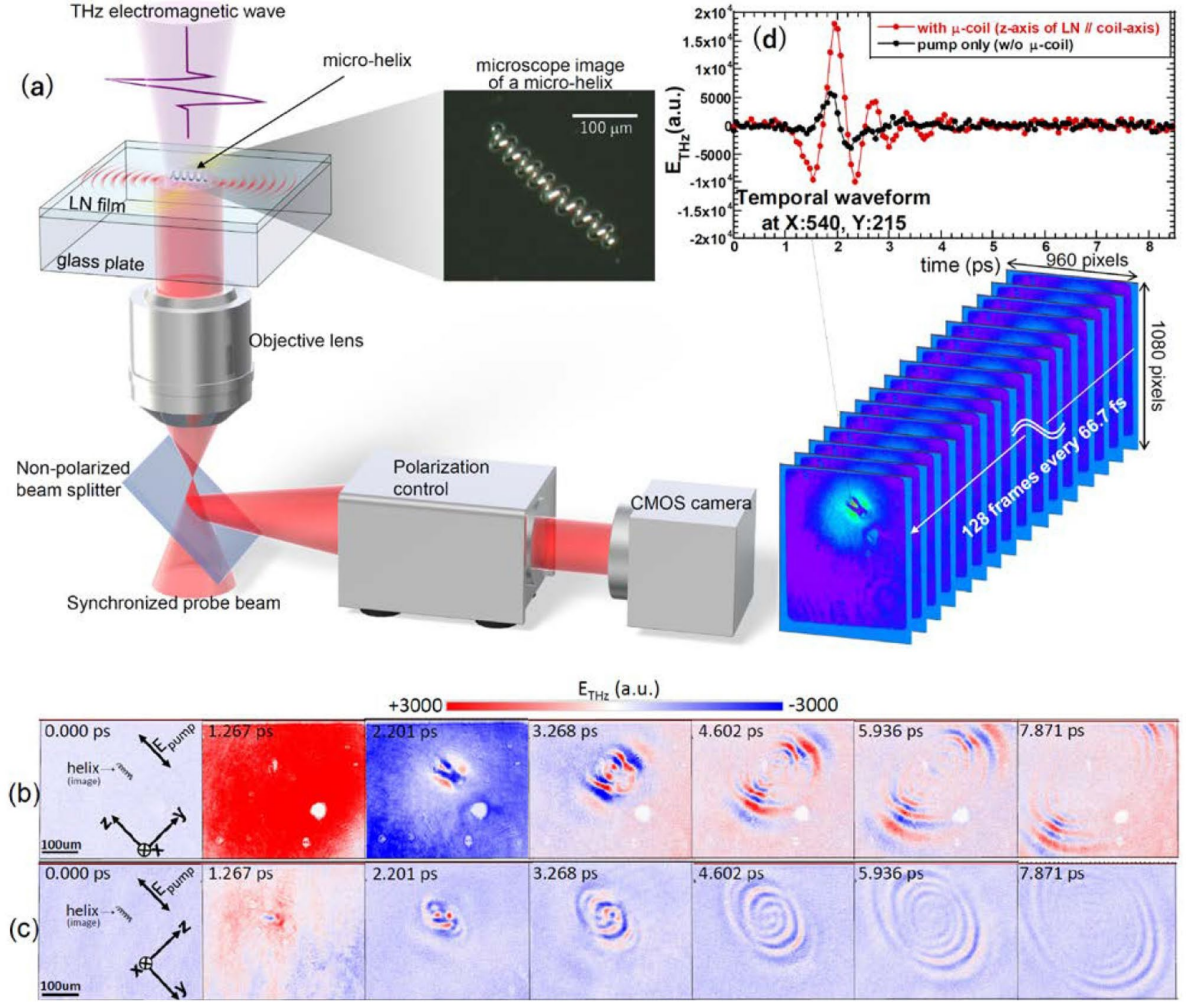
THz near-field microscopy for a metal micro-helix. (**a**) Schematic illustration of THz near-field microscopy for a single nickel-coated micro-helix obtained from a biological *Spirulina* template. The micro-helix is attached directly to a thin LiNbO_3_(LN) crystal film. The sensitive crystal-axis direction for THz electric fields depends on the configuration of the LN crystal. The change in the refractive index of the LN crystal due to the transmission and re-emission of THz electric fields changes the polarization state of the probe beam, which is converted into an intensity change and captured by a CMOS camera. (**b**) and (**c**) Several temporal snapshots of the near-field electric-field distributions of transmitted and re-emitted THz electromagnetic waves. Here, (**b**) and (**c**) correspond respectively to the evolution of radiation perpendicular and parallel to the helix axis, and there is only a 90° difference in the direction of the LN crystal axis. In the snapshots at 0 ps, a representation of the micro-helix is shown for reference. These snapshots clearly show that impulsive THz electromagnetic-wave irradiation of the micro-helix is accompanied by dynamical anisotropic re-emission. The temporal and spatial resolution of the microscopy are 66.7 fs and 14 μm, respectively. (**d**) Representative temporal waveforms of the THz electric fields at a point beneath the approximate center of the micro-helix. First, the THz pump pulse, with roughly a 3 ps pulse width, irradiates the micro-helix and penetrates the thin LN crystal, and then re-emission from the micro-helix begins to overlap it. The red and black dotted lines show the temporal waveforms with and without the micro-helix, respectively.

A schematic image of the process of near-field THz microscopy for a metal micro-helix is shown in Fig. 1a. Here, the near-field THz microscope is mainly developed by co-authors (T. Arikawa and K. Tanaka) and detailed description of the system is already given^10–12^. Figure 1b,c shows the experimental results for the temporally evolving THz electrical fields, including their interactions with the 3D metal micro-helix. Seven illustrative snapshots have been extracted from movie-1 (link1) and movie-2 (link2), which consist of 128 flames recorded every 66.7 fs during the total time of 8.5 ps. The field of view of each snapshot corresponds to a 400 × 344 μm square. In Fig. 1b, the helix axis and the polarization of the incident THz wave are oriented parallel to the z-axis of the LiNbO_3_ (LN) crystal, as shown in the snapshot at 0 ps. Our setup mainly detects the electric field component polarized along the z-axis, while the sensitivity for the y-component is low due to the inequality between the electro-optic coefficients of the LN crystal (r33 ≫ r22). The pumping THz waves reach the top surface of the LN crystal at around 0.6 ps, and THz waves re-emitted from the helix can be perceived to start expanding after roughly 2.0 ps. The re-emission broadens around the helix, and the flat wavefront directs the propagation perpendicular to the helix axis (perpendicular re-emission). Consequently, the radiation is transverse to the longitudinal helix axis and matches dipole radiation.

On the other hand, a distinct re-emission pattern is observed with a different setup, which is provided simply by rotating the LN crystal by 90 degrees while keeping other conditions unchanged. In this configuration, the direction of polarization of the incident THz wave and the helix axis remain parallel, but they become perpendicular to the z-axis of the LN crystal, as shown in the snapshot at 0 ps in Fig. 1c. Hence, a THz electric field polarized perpendicular to the helix axis is effectively detected. The results show that re-emission occurs along the axial direction of the micro-helix (parallel re-emission), with a spirally expanding wavefront that is remarkably curved because the radiative aperture is smaller than the wavelength. In this way, two anisotropic re-emissions occur simultaneously from a single metal micro-helix in response to impulsive THz-wave irradiation. According to this direct measurement of the interaction between a single micro-helix and a THz electromagnetic wave, the strong attenuation of the THz wave in a paraffin sheet that contains micro-helices as inclusions^9^ must be caused by multiple chain-reaction processes. During those processes, an individual micro-helix alternates between receiving and emitting THz-wave energy, re-absorbing and re-emitting radiation from other surrounding micro-helices, and the THz-wave energy is gradually dissipated, mainly due to radiative and ohmic losses.

**Figure 2:**
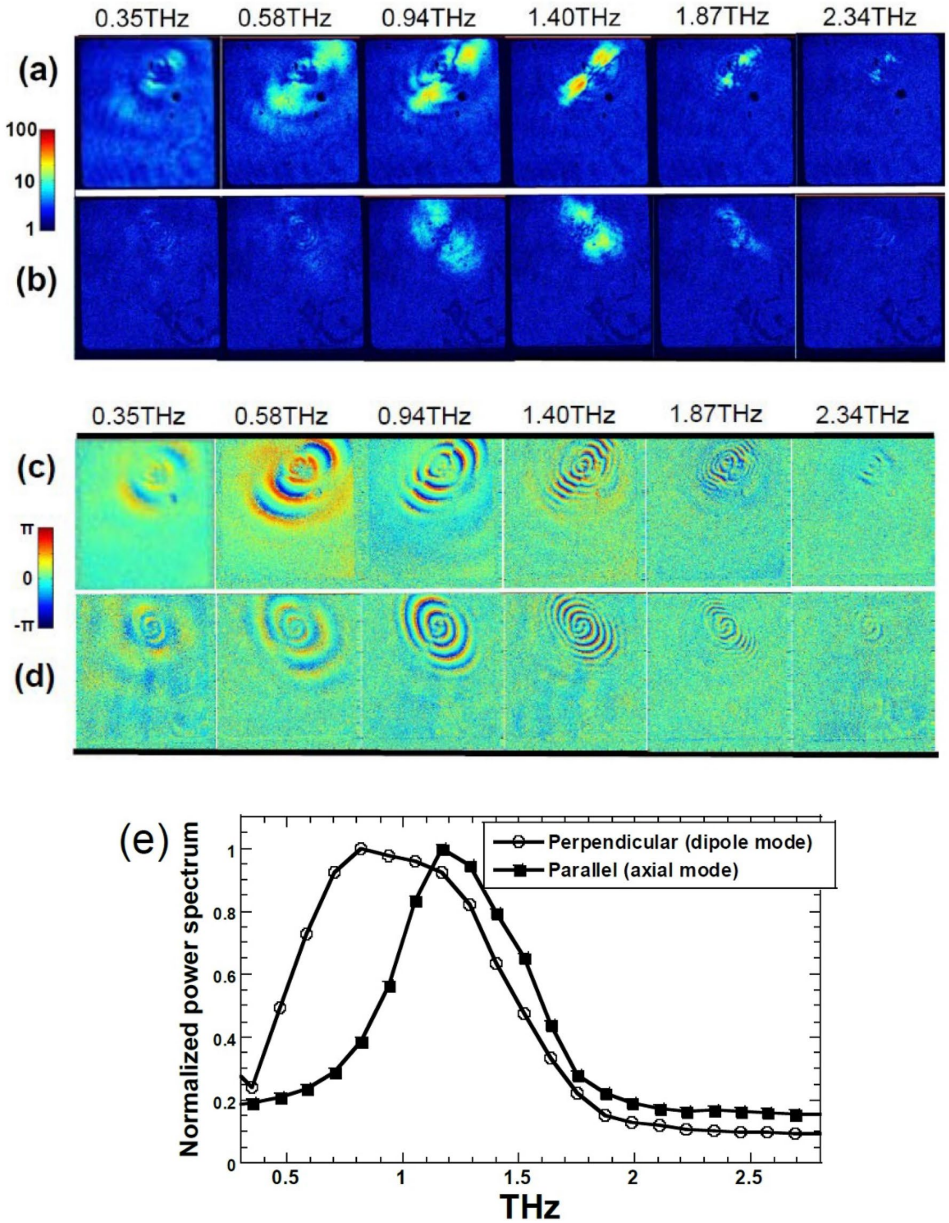
Fourier-transformed data retrieved from the dynamical time evolution of THz electric fields. The visualized amplitude distributions of the THz electric fields correspond to (**a**) perpendicular and (**b**) parallel re-emissions with respect to the helix axis at several THz frequencies. The amplitude color bar corresponds to a logarithmic scale. Panels (**c**) and (**d**) show the visualized phase distributions of the THz electric fields corresponding to (**c**) perpendicular and (**d**) parallel re-emissions at several THz frequencies. The phase color bar corresponds to a linear scale. In these four figures, each image is a 550 × 620 μm square, and the frequency resolution is approximately 100 GHz. (**e**) Spatially averaged power spectra. Perpendicular re-emissions are represented by circles and parallel re-emissions by squares. The time-domain data are first Fourier transformed at each pixel. Then, the frequency-spectrum data are averaged spatially at each frequency and normalized by the maximum values.

Figure 1d shows an example of the temporal waveforms of the THz electric fields obtained beneath the approximate center of a micro-helix. The measurement was originally done in the time domain; therefore, spectroscopic information at each pixel can be obtained by Fourier transforming the temporal waveform. The first giant oscillation given by the red dotted line is mainly from the irradiating THz-wave pulse, and the re-emitted THz electric field becomes dominant after 3 ps. Figure 2a,b corresponds to the Fourier-transformed amplitude distributions at several representative THz frequencies for the perpendicular and parallel re-emissions, respectively. The difference in active frequency between the two re-emissions can be seen explicitly; that is, the perpendicular and parallel re-emissions are effectively radiated at lower and higher THz frequencies, respectively. In order to make the difference in the frequency spectra clear, the Fourier-transformed 2D amplitudes are spatially averaged at each frequency, and the result is shown in Fig. 2e. The peak frequency of the perpendicular re-emission occurs at approximately 0.8 THz, which corresponds to the peak frequency of the broadband pump THz-wave pulse. On the other hand, the peak frequency of the parallel re-emission occurs at approximately 1.2 THz, which is shifted noticeably toward higher frequencies. In Fig. 2b, the parallel re-emission is not observed so clearly at frequencies exceeding 2 THz. This is because the original pumping THz-wave pulse has a maximum spectrum amplitude at around 0.8 THz, and higher THz frequency components are relatively weak. In analyzing Fig. 2 to eliminate the influence of the intrinsic frequency spectrum of the pumping THz-wave pulse as much as possible, we performed Fourier-transform calculations for all time-domain data except for the first 3 ps, where the pumping THz-wave pulse is dominant compared to re-emission from the micro-helix, as already shown in Fig. 1d. However, the frequency characteristics of these distinct re-emissions are still influenced by the original frequency spectrum of the pumping THz-wave pulse, because the re-emissions always inherit the spectrum of the pump pulse through alternating-current (AC) excitation in the helix. In order to evaluate the genuine frequency characteristics of a micro-helix, it is necessary to perform convolution integrations between the pumping THz electric-field function and the impulse-response function in the Fourier domain.

In addition to the amplitude, phase information also is very valuable, because it governs wave-quality parameters such as coherence, the polarization state, and the direction of propagation. In contemporary imaging and microscopy technologies, phase information usually provides superior sensitivity, resolution, and dynamic range as compared to amplitude information. Furthermore, phase information can substantially improve the synthesized antenna performance, as recently demonstrated in the first imaging of a black hole in the universe^13^. However, direct measurements of the real-time evolution of the phase distributions of electromagnetic waves are extremely difficult to obtain with conventional technologies. Our approach does make it possible to visualize the dynamical evolution of the phase distributions of THz electromagnetic waves. Figure 2c,d shows the Fourier-transformed phase distributions for perpendicular and parallel re-emissions, respectively. The curvature radius of the phase front appears to be smaller for parallel re-emission than for perpendicular re-emission. Therefore, a parallel re-emission wave will spread more rapidly due to stronger diffraction, because the aperture of the radiative source is relatively narrow due to the elongated helix structure. Such phenomena cannot be understood from amplitude data alone, and this effectively demonstrates the importance of real-time phase-distribution measurements. Although the amplitude profiles for parallel re-emissions at 0.35 and 0.58 THz are very faint, as shown in Fig. 2b, their corresponding phase profiles can be clearly captured, as shown in Fig. 2d.

The anisotropic, frequency-decomposed re-emissions can be understood by analogy with antenna theory^14^. Parallel re-emission results in axial-mode radiation, which is a characteristic radiation pattern from a helical antenna that is widely used in GPS and mobile communication systems. Perpendicular re-emission corresponds to dipole-mode radiation, which is effectively observed at lower THz frequencies in our measurements. Because even a 3D helix can be regarded as a simple straight rod when the total wire length of the helix is much shorter than the corresponding THz wavelength, dipole emission is also possible at lower THz frequencies. The influence of helix dimensions, such as pitch angle, helix diameter, numbers of turn and length, on these re-emission patterns or frequency response is an interesting topic, and it will be measured and reported in the near future.

Since our measurements are based on impulsive THz-wave excitation, the wideband frequency characteristics of the micro-helix must be known. We have therefore carried out eigenmode simulations with the finite-element method using ANSYS HFSS software in order to determine whether the resonant vibration frequencies of a micro-helix really exist in the THz frequency range. We used the helical parameters length = 120 μm, coil diameter = 23 μm, wire diameter = 7 μm, pitch angle = 17°, and number of turns = 6 to simulate the micro-helix used in the measurement. In the simulation, the metal is assumed to be a perfect conductor in order to ignore skin-depth effect and ohmic loss for simplification. The external boundary condition is set to be a perfectly matched layer^15^, which produces zero reflections at the boundary surfaces in 3D simulation space. The simulation results showed the resonant vibration frequencies to be 0.36, 0.65, 0.90, 1.13, 1.36, 1.62, 1.85, and 2.11 THz for the modeled micro-helix structure. We performed the simulations over the limited frequency range from 0.1 to 2.5 THz, which is equivalent to the bandwidth of the pumping THz-wave pulse of the THz near-field microscope. The quality factors (Q-values) corresponding to each resonant frequency are 9.2, 17.1, 24.9, 43.4, 72.9, 70.1, 54.6, and 10.9, respectively, from the simulation. The maximum Q-value is obtained at a resonant frequency of approximately 1.36 THz, which is in reasonable agreement with the measurement results. Such high Q-values may not be obtained for an actual micro-helix due to deviations from an ideal helical structure. In addition, there also may be some differences between the helical parameters of the ideal simulated shape and real ones. Further, as mentioned above, the observed re-emission frequency characteristics are strongly influenced by the original spectrum of the pumping THz pulse, which is not considered in this eigenmode analysis.

The THz near-field microscope is the only tool that can directly visualize dynamical THz electromagnetic-wave behavior in real time and in real 2D space. We have performed simulations in order to understand better the temporal and spatial evolution of the electromagnetic waves and the frequency characteristics of various objects. However, simulation results at ultra-high frequencies exceeding 300 GHz are not so reliable, because the complex permittivity and conductivity of materials in this region have not yet been characterized accurately. Even a slight deviation of a 3D structure from the ideal may cause significant malfunctions in THz devices. Therefore, it is very important to measure directly the real behaviors of THz electromagnetic waves, including complex interactions with actual 3D objects as demonstrated in this paper, because the utilization of THz electromagnetic waves is becoming increasingly important for the next-generation wireless telecommunication society.

## Discussion and conclusions

We have succeeded in measuring the real-time evolution of THz electric-field distributions, including intricate interactions with a single 3D metal micro-helix. Spatially anisotropic, frequency-decomposed re-emissions from the micro-helix in response to impulsive THz-wave irradiation are visualized dynamically. Although they are analogous to dipole- and axial-mode radiation in terms of the operation of a helical antenna, such dynamical, transient behaviors of a metal micro-helix have not been measured before. Direct visualization is much more informative than simulations; therefore, our findings will be particularly valuable for research and development on various electromagnetic devices that deal with THz electromagnetic waves. For example, frequency-division multiplexing/demultiplexing capabilities^16^ and directional adaptivity^17^ are key technologies required for smart antennas to support advanced wireless telecommunication networks. Anisotropic, bidirectional emissions with differently decomposed frequency spectra from the micro-helix, as demonstrated here, can satisfy such requirements. In addition, the broadband characteristics and the ability of helical antennas to emit and receive both linear and circular polarizations are very attractive for achieving ultrafast wireless mobile telecommunications^18^. Conventionally, antenna-performance characteristics such as directivity and gain have been studied by measuring far-field radiation patterns while scanning a probe point-by-point. However, the probe itself causes unwanted standing waves and disturbs the actual electromagnetic fields to be measured. Our approach permits measurements of the actual amplitude and phase distributions more accurately and without such field perturbations or probe scanning. Furthermore, although antennas are usually operated at a single AC frequency fed by impedance-matched CW power, studies of the impulsive responses of antennas and/or related devices have become important recently. Because of the increasing numbers of wireless telecommunication devices and the background of impulsive noise radiation that surrounds us, understanding the operating characteristics of devices in such noisy environments is important for constructing more reliable wireless mobile networks using beyond-5G platforms. As the carrier frequencies of wireless electromagnetic waves increase, the sizes of the corresponding antennas and devices become smaller. Consequently, both the performance evaluation and the fabrication of such devices become more difficult. Bio-templating processes enable mass production of metal micro-helices at very low cost, compared to other methods such as lithography and 3D printers^9^. Although Gansel has used a commercial 3D printer with direct laser writing to fabricate a μm-scale helical array^19^, 3D printing is incredibly inefficient, even for laboratory-use prototypes, costing time and money, and it would be out of the question for industrial production.

Our results also suggest other interesting topics. First, a metal micro-helix also can be regarded as a 3D meta-atom, which is an element of a metamaterial. Generally, the electromagnetic responses of metamaterials have been studied by measuring the macroscopic transmittance and reflectance. The interaction between a single 3D meta-atom and electromagnetic waves has scarcely been studied experimentally to understand the metamaterial behavior of electromagnetically coupled meta-atoms. Our results may be the first measurement of the direct microscopic response of a single 3D meta-atom. By using a THz near-field microscope, the behavior of electromagnetic coupling with multiple surrounding meta-atoms can be measured, which provides a more profound understanding of functioning of advanced metamaterials. Second, the physics of the neighborhood electromagnetic fields of antennas are not understood, because even in the long history of electricity and magnetism only far-fields have been studied. However, neighborhood fields—such as quasi-static fields and inductive fields^20^ that exist only in the vicinity of a radiation source—contain essential information about electromagnetic-wave creation. Such non-radiative neighborhood fields have recently been attracting much attention for wireless power transfer^21^, vicinity high-resolution sensing^22^, biometrics^23^, and electromagnetic compatibility studies^24^. Since our measurements also contain neighborhood electric fields, further studies may provide new information about the behavior of such non-radiative fields.

## Methods

The transient electromagnetic responses of a metal micro-helix to impulsive THz-wave excitation are visualized using a THz near-field microscope^10–12^, which is a cutting-edge tool that directly and instantaneously measures THz electric fields with high spatial resolution. A schematic of the experimental setup is shown in Fig. 1a. Intense THz-wave pulses with 3 ps pulse widths are generated by using a tilted-pulse-front technique^25^ (outside the frame of the figure). They are focused on a micro-helix that is placed on an x-cut LiNbO_3_ (LN) crystal film deposited on a glass plate. The spot size and electric-field intensity are approximately 500 μm and 200 kV/cm, respectively. The thicknesses of the LN and the glass plate are 10 μm and 500 μm, respectively, with a 5 × 5 mm aperture. The 2D electric fields of the THz waves are sampled with the LN crystal by means of the electro-optic (E-O) effect by using a probe beam with a pulse duration of 100 fs at a wavelength of 780 nm. The temporal changes of the electric fields are obtained by synchronizing the timing of the probe-beam irradiation with the incident THz waves. The probe beam is coupled from the backside of the glass plate through an objective lens and a non-polarized beam splitter, and it is reflected by a thin high-reflection coating applied between the helix and the LN crystal. The coating is too thin to be affected by the THz waves. The polarization state of the probe beam is modulated during round-trip propagation in the LN crystal. The beam passes back in the direction of the beam splitter and is partially reflected to a polarization analyzer, where it is converted into an intensity-modulated signal. The signal image is then captured by a CMOS camera using a balanced imaging scheme to achieve a high S/N ratio. The CMOS sensor has 960 × 1080 pixels which covers a physical area of 550 × 620 μm^2^. The high spatial resolution of 14 μm (corresponding to λ/30, based on a center frequency of 0.7 THz) is confirmed^12^; it is over the diffraction limit owing to the shorter wavelength of the probe beam and immediate interactions within the thin LN crystal.

To obtain a metal micro-helix, a sample of *Spirulina*, serving as a biotemplate, is nickel-coated. The thickness of the coating is typically several hundred nm, which is much thicker than skin depth for a THz wave. For the measurements, the sample is placed directly onto the LN crystal and is fixed there using Scotch tape, which is almost transparent to THz waves. Intense THz-wave pulses irradiate the helix almost perpendicularly from above, and their polarization is linear and parallel to the helix axis. Since the measurement of the electrical field depends sensitively on the LN crystal axis, the sample is rearranged by rotating the crystal with respect to the polarization of the incident electrical field. The length and diameter of the helix, the wire diameter, and the pitch angle of the sample micro-helix are approximately 120 μm, 23 μm, 7 μm, and 17°, respectively.

## Supplementary Information

Supplementary Information.

Supplementary Information.

Supplementary Information.
