# Supplementary material for: Dynamical visualization of anisotropic electromagnetic re-emissions from a single metal micro-helix at THz frequencies
Source: Sci Rep. 2021 Feb 8;11:3310. doi: 10.1038/s41598-020-80510-y (PMC7870654; doi:10.1038/s41598-020-80510-y)
Supplement: Supplementary file 1 — Supplementary Information. [file 41598_2020_80510_MOESM1_ESM.pdf]

Two movies are very important to visually understand importance of this manuscript. We uploaded .avi-files as supplemental files at following links. Names of files correspond to the names written in the result section of the manuscript.

Movie-1

https://briefcase.riken.jp/public/37wUgAIZM4DA_uo

Movie-2

https://briefcase.riken.jp/public/p7BQgAGZUEDAfg0

Title:

Dynamical visualization of anisotropic electromagnetic re-emissions from a single metal micro-helix at THz frequencies

Author list

T. Notake[1*], T. Iyoda[2], T. Arikawa[3], K. Tanaka[3], C. Otani[1], H. Minamide[1]

[1]Center for Advanced Photonics, RIKEN, 519-1399, Aramaki-aza Aoba, Sendai 980-0845, Japan.

[2]Harris Science Research Institute, Doshisha University, 1-3 Tatara Miyakodani, Kyotanabe, Kyoto 610-0394, Japan.

[3]Department of Physics, Graduate School of Science, Kyoto University, Kitashirakawa Oiwake-cho, Sakyo-ku, Kyoto 606-8502, Japan.

Corresponding author; notake@riken.jp
